# The short and long term effects of exercise training in non-cystic fibrosis bronchiectasis – a randomised controlled trial

**DOI:** 10.1186/1465-9921-15-44

**Published:** 2014-04-15

**Authors:** Annemarie L Lee, Catherine J Hill, Nola Cecins, Sue Jenkins, Christine F McDonald, Angela T Burge, Linda Rautela, Robert G Stirling, Philip J Thompson, Anne E Holland

**Affiliations:** 1 Alfred Health, Commercial Road, Melbourne 3004, Victoria, Australia; 2 Institute for Breathing and Sleep, 145 Studley Road, Heidelberg 3084, Australia; 3 Physiotherapy, Melbourne School of Health Sciences, The University of Melbourne, 161 Barry Street, Carlton 3010, Australia; 4 Austin Health, 145 Studley Road, Heidelberg 3084, Australia; 5 Sir Charles Gairdner Hospital, Hospital Avenue, Nedlands, Perth 6009, Australia; 6 Lung Institute of Western Australia and Centre for Asthma, Hospital Avenue, Nedlands, Perth 6009, Australia; 7 Curtin University, Kent Street, Bentley, Perth 6102, Australia; 8 Department of Medicine, Monash University, Melbourne 3800, Australia; 9 Physiotherapy, La Trobe University, Melbourne 3086, Australia

**Keywords:** Bronchiectasis, Exercise training, Quality of life, Exercise capacity, Acute exacerbations

## Abstract

**Background:**

Exercise training is recommended for non-cystic fibrosis (CF) bronchiectasis, but the long-term effects are unclear. This randomised controlled trial aimed to determine the effects of exercise training and review of airway clearance therapy (ACT) on exercise capacity, health related quality of life (HRQOL) and the incidence of acute exacerbations in people with non-CF bronchiectasis.

**Methods:**

Participants were randomly allocated to 8 weeks of supervised exercise training and review of ACT, or control. Primary outcomes of exercise capacity and HRQOL (Chronic respiratory disease questionnaire) and secondary outcomes of cough-related QOL (Leicester cough questionnaire) and psychological symptoms (Hospital anxiety and depression scale) were measured at baseline, following completion of the intervention period and at 6 and 12 months follow up. Secondary outcomes of the exacerbation rate and time to first exacerbation were analysed over 12 months.

**Results:**

Eighty-five participants (mean FEV_1_ 74% predicted; median Modified Medical Research Council Dyspnoea grade of 1 (IQR [1–3]) were included. Exercise training increased the incremental shuttle walk distance (mean difference to control 62 m, 95% CI 24 to 101 m) and the 6-minute walking distance (mean difference to control 41 m, 95% CI 19 to 63 m), but these improvements were not sustained at 6 or 12 months. Exercise training reduced dyspnoea (p = 0.009) and fatigue (p = 0.01) but did not impact on cough-related QOL or mood. Exercise training reduced the frequency of acute exacerbations (median 1[IQR 1–3]) compared to the control group (2[1–3]) over 12 months follow up (p = 0.012), with a longer time to first exacerbation with exercise training of 8 months (95% CI 7 to 9 months) compared to the control group (6 months [95% CI 5 to 7 months], p = 0.047).

**Conclusions:**

Exercise training in bronchiectasis is associated with short term improvement in exercise capacity, dyspnoea and fatigue and fewer exacerbations over 12 months.

**Trial registry:**

ClinicalTrials.gov (NCT00885521).

## Introduction

Bronchiectasis unrelated to cystic fibrosis (non-CF bronchiectasis) is a chronic respiratory condition characterised by bronchial dilatation secondary to airway inflammation, infection and dysfunction of mucociliary clearance [1]. Common features include persistent cough with chronic sputum production, dyspnoea and fatigue [2,3]. This clinical profile is associated with increased anxiety and depression, reduced health-related quality of life (HRQOL) and impaired exercise tolerance [3,4]. Patients with non-CF bronchiectasis frequently experience acute exacerbations [5], which are an independent predictor of progressive decline in respiratory function and a poorer prognosis [6,7]. While the global prevalence of non-CF bronchiectasis is unknown, it is associated with rising hospitalisation rates amongst the older population [8-10]. This growing healthcare utilisation and economic burden [11,12] emphasises the need for effective medical and physiotherapy treatment approaches.

Current guidelines for the treatment of non-CF bronchiectasis recommend pulmonary rehabilitation, with the aim of improving exercise tolerance and HRQOL [13]. Pulmonary rehabilitation often incorporates self-management strategies to promote treatment adherence and this approach has been advocated for bronchiectasis [14]. There is limited evidence of the effects of pulmonary rehabilitation in non-CF bronchiectasis, with two retrospective studies demonstrating similar benefits in exercise capacity and HRQOL as those observed in patients with chronic obstructive pulmonary disease (COPD) [15,16]. Two prospective studies showed short-term improvements in exercise capacity and HRQOL with a combination of endurance, strength training and inspiratory muscle training [17] or regular airway clearance therapy (ACT) [18]. These studies incorporated formal education as part of the clinical approach and had a follow up period of three months; it is not clear if benefits persist in the longer term.

Health benefits are also assessed through quality-adjusted life years (QALYs), which quantify the benefit gained from an intervention by measuring the change in HRQOL over time. In COPD, pulmonary rehabilitation (exercise training and education) has been associated with fewer acute exacerbations and improved QALYs [19]. Whether similar effects are achieved in bronchiectasis is unknown.

Therefore, the aims of this study were to 1) determine the short and long-term effects of exercise training and review of ACT on exercise capacity and dimensions of HRQOL and 2) evaluate the effect of this intervention on the incidence of acute exacerbations over a 12-month follow up period.

## Methods

### Participants

A multi-site, randomised, single blinded, controlled trial was conducted at three tertiary teaching hospitals, the Alfred and Austin Hospitals (Victoria) and Sir Charles Gairdner Hospital (Western Australia), Australia. Adults aged over 18 years with non-CF bronchiectasis, confirmed on high resolution computed tomography (HRCT), were invited to participate. The detailed methodology of this study has been published previously [20].

Patients were eligible to participate if they were ambulant and reported dyspnoea on exertion (Modified Medical Research Council (MMRC) Dyspnoea grade ≥ 1) [21], clinically stable and self-reported at least two exacerbations requiring antibiotics per year within the previous two years. Exclusion criteria were other concurrent respiratory diseases, including COPD (smoking history of greater than 10 pack years and evidence of emphysema on HRCT) [22], interstitial lung disease (ILD) or asthma (clinical diagnosis and reversibility > 12%) [23], comorbidities which precluded safety to undertake exercise training or participation in pulmonary rehabilitation in the previous 12 months. The study was approved by the Human Research Ethics Committees at all trial sites with written informed consent obtained from all participants. The trial was registered with ClinicalTrials.gov (NCT00885521).

After stratification for sputum quantity (small volume: ≤one tablespoon per day, large volume: > one tablespoon per day), which was based on classifications previously described [2] and is associated with decline in FEV_1_[7], participants were randomised to the intervention or control group at a central location using a computer-generated randomisation sequence, which was concealed using sealed, opaque envelopes and prepared by an independent individual. Baseline measurements of spirometry were obtained and repeated immediately following the intervention period and at follow up. Prior to randomisation, all participants received instruction and review of their usual ACT, with instructions standardised across sites. Any participant who had no prior instruction in ACT was taught the active cycle of breathing technique [24] while those with established ACT had their technique reviewed and corrected if necessary. All participants, irrespective of group allocation, were encouraged to continue with their ACT for the study duration.

### Intervention

The intervention group attended a twice-weekly exercise program for eight weeks at the institution in which they were recruited. Exercise training consisted of an individually prescribed exercise program and included treadmill or land based walking, with the initial intensity set to 75% of the maximal speed achieved on the incremental shuttle walk test (ISWT) [25,26], stationary cycling prescribed at 60% of maximal work-rate for cycling [27] and upper and lower limb strength training using free weights and/or body weight [25,26]. Exercises were progressed each session according to patient symptom ratings (modified Borg scale for dyspnoea and perceived exertion), similar to COPD training principles [25,26]. Attendance of at least 12 out of 16 exercise sessions was considered completion of the exercise intervention [28]. Following the establishment of a safe exercise regimen, a home exercise program was prescribed in week one, with the aim of achieving three to five unsupervised sessions per week with sessions recorded in an exercise diary. Participants were instructed to maintain this routine during the follow up period and were reminded via monthly telephone calls over the follow up period. To encourage adherence to ACT, an average rating of sputum volume using a visual analogue scale (VAS) was recorded weekly. If necessary, ACT was reviewed and corrected.

The control group did not receive supervised exercise training but were informed at baseline that undertaking 30 minutes of moderate intensity physical activity most days of the week was associated with health benefits [29]. During the eight week intervention, participants were contacted by telephone twice-weekly, to provide support and general advice with no discussion of exercise or physical activity. The control group did not complete an average sputum rating and did not have an opportunity for their ACT to be reviewed or corrected.

### Outcome measures

Over two days of assessment at each time point, primary and secondary outcomes were measured at baseline, immediately following the intervention (nine weeks) and at six and 12 months post intervention by an assessor blinded to group allocation. One site did not participate in the long term follow up due to ethical concerns relating to denial of exercise training to the control group for 12 months. The primary outcome of maximal exercise capacity was the incremental shuttle walk distance (ISWD), with the ISWT performed to a standardised protocol [30]. Two tests were completed at each assessment, with the maximal distance of two tests recorded [30]. The self-reported version of the Chronic respiratory disease questionnaire (CRDQ) was used to measure HRQOL [31]. This has been previously used to assess HRQOL in patients with bronchiectasis [15].

Secondary outcomes included functional exercise capacity, measured with the 6-minute walk test (6MWT). Two tests were completed on each assessment occasion to a standardised protocol, with the maximal 6-minute walk distance (6MWD) of the two tests recorded [32]. The Leicester cough questionnaire (LCQ) was applied to measure the physical, psychological and social impact of chronic cough [33] and the Hospital anxiety and depression scale (HADS) measured psychological symptoms of anxiety and depression [34].

To determine an individual’s utility of health status at a specific point in time, the Assessment of Quality of Life (AQOL) was applied [35]. The AQOL has five dimensions including illness, independent living, social relationships, physical senses and psychological well-being, with item responses based on four point ordinal scales. The AQOL was converted into a value index using preference weights derived from Australian population samples, which were combined into a single score of the utility index [35]. The highest score of 1 represents perfect health, 0 represents death and negative values (i.e. -0.04) represent a state of worse than death. The health state was then multiplied by the time spent in that state (one year in this study) to derive the QALYs gained or lost following an intervention [19,35].

During the eight week intervention period and over the 12 month follow up, all participants maintained a daily diary recording changes in symptoms. This symptom record was used to identify an exacerbation, which was defined as the presence of ≥ four signs and symptoms (including change in sputum amount, thickness or colour, haemoptysis, increased cough, tiredness, shortness of breath or fever> 38 degrees Celcius) [5] for two or more consecutive days with and without prescription of new antibiotics. To ensure adherence to diary completion, participants in both groups were contacted by telephone monthly over the follow up period. Exacerbation data were extrapolated from diary records by an independent assessor blinded to group allocation, which was then verified with the participant’s general practitioner or hospital records.

### Statistical analysis

For the primary outcomes, for an 80% probability of detecting a difference in ISWD, a total of 36 participants were required, based on the assumption of a difference between groups of 55 m, with a standard deviation (SD) of 74 m [3]. For an 80% probability of detecting a difference in HRQOL, 44 subjects were required. This was based on the assumption that a difference between groups is 5.4 units for the CRDQ emotional function domain, with a SD of 6.3 units using an unstandardised scale, according to pilot data collected on 33 patients at one recruiting centre. For the secondary outcomes, for an 80% probability of detecting a difference in number of exacerbations, 64 participants were required, based on the assumption that a difference between groups was 1.0 exacerbation, with a SD of 1.4 exacerbations [7]. To account for 20% attrition, a total of 85 subjects were recruited.

**Figure 1:**
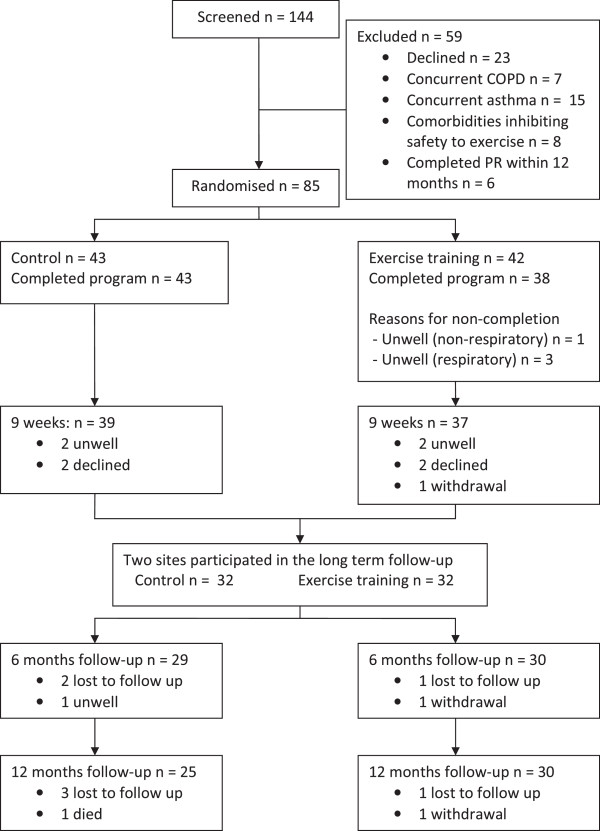
Flow of participants.

Data analysis was completed based on intention-to-treat principles, in which all participants were scheduled to attend follow up assessment, irrespective of completion and all available data from participants attending these assessment (Figure 1) has been included in the analysis [36]. Missing data were replaced by the last observation carried forward method (LOCF), with the robustness of this analysis checked with a sensitivity analysis in which the observed means from the exercise and control groups were substituted for missing data in the opposite arm [37]. The sensitivity analysis confirmed the results of the primary endpoint and hence LOCF analyses have been reported. Data were expressed as mean and standard deviation (SD) or median and interquartile range (IQR). Analysis was completed using two way analysis of variance to account for group and time interaction. Non parametric data were compared between groups using the Mann Whitney U test. Time to first exacerbation was assessed via Kaplan-Meier survival analysis using log-rank tests to compare groups. Analyses were performed using the Statistical Package for Social Sciences (SPSS version 17.0; Chicago, IL, USA) with p < 0.05 used to denote statistical significance.

## Results

Between April 2009 and February 2011, a total of 144 patients were screened (Figure 1), 36 were excluded prior to randomisation for not meeting the inclusion criteria and a further 23 declined to participate. The subject flow reflected the recommendations from the Consolidated Standards of Reporting Trials (CONSORT) statement [38]. A total of 42 were randomised to the exercise training group and 43 to the control group. All participants had been previously prescribed ACT. For those in the intervention group, no changes to ACT regimens were required by any participant over the eight week duration and the average VAS sputum volume rating did not change over the intervention period (p = 0.56). Adherence to exercise diary completion during the 8 week program in the exercise group was 29%. All control group participants reported no change to their usual exercise patterns between baseline assessment and at 9 weeks follow up.

One participant in the exercise group withdrew from the study during the intervention phase due to an illness unrelated to bronchiectasis. Data for this participant were not available at nine weeks, six or 12 months. There was one death in the control group from respiratory failure. A total of 38 (90%) participants completed the exercise training, with reasons for non-completion outlined in Figure 1. Reasons for not undertaking outcome measures at the nine week assessment were respiratory illness (n = 3), fractured thoracic spine which was unrelated to exercise training (n = 1) or declined to attend (n = 4). Baseline characteristics of the 85 participants are shown in Table 1, with no difference between groups at baseline in any measures. According to the MMRC Dyspnoea grade, the extent of functional impairment in exercise capacity was mild with 64 (75%) participants reporting dyspnoea only on strenuous activity (MMRC Grade 1). There was no change in FEV_1_ (p = 0.26) or FVC% predicted (p = 0.43) over the study duration in either group.

**Table 1:** Baseline demographics of participants

	**Control n = 43**	**Exercise n = 42**
Gender: Male/Female	12/31	12/30
Age (years)	65 (12)	63 (13)
BMI (kg/m^2^)	24.2 (4.5)	24.7 (5.9)
FEV_1_% predicted	77 (18)	70 (23)
FVC% predicted	84 (18)	79 (22)
FEV_1_/FVC	80. (14)	82 (12)
MMRC	1.5 (0.7)	1.3 (0.5)
6MWD (m)	578 (90)	551 (90)
6MWD% predicted	84 (12)	85 (12)
ISWD (m)	474 (157)	464 (150)
ISWD% predicted	77 (22)	76 (24)
Total CRDQ score	91.0 (18.6)	88.9 (16.1)
Total LCQ score	14.5 (3.6)	15.1 (3.1)
HADS Anxiety	4.4 (3.1)	4.8 (3.4)
HADS Depression	3.5 (2.8)	3.1 (2.9)
No of exacerbations in previous 2 years	5.3 [4.8]	5.1 [4.6]

Data are mean (SD) or median [IQR] unless otherwise stated. BMI - Body mass index; FEV_1_ - Forced expiratory volume in 1 second; FVC - Forced vital capacity; MMRC – Modified Medical Research Council Dyspnoea Scale; 6MWD – 6-minute walk distance; ISWD – Incremental shuttle walk distance; CRDQ – Chronic Respiratory Disease Questionnaire; LCQ – Leicester Cough Questionnaire; HADS – Hospital Anxiety and Depression scale; No – number.

**Figure 2:**
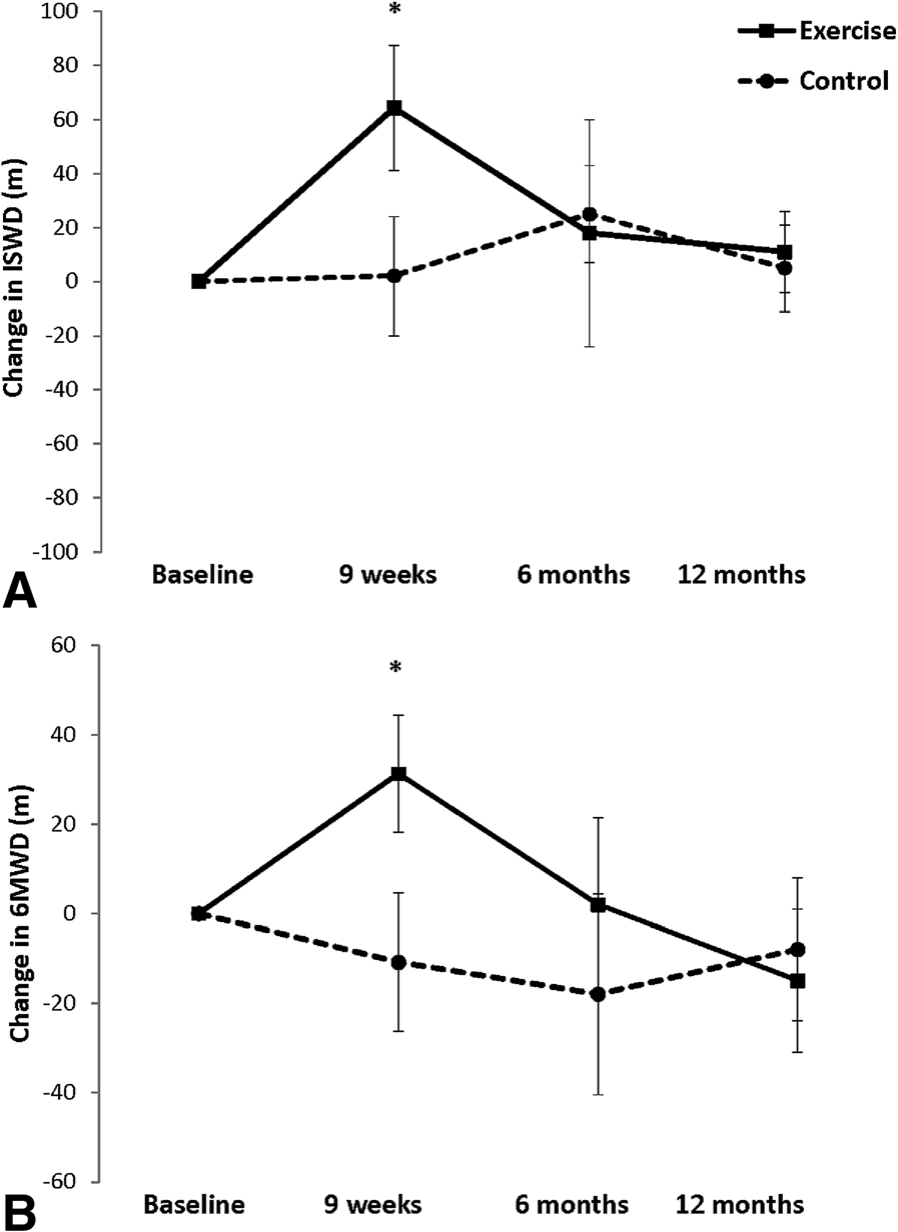
Change in incremental shuttle walk distance (A) and 6-minute walk distance (B). Data are mean (95% CI), *p < 0.05, exercise vs control group.

**Figure 3:**
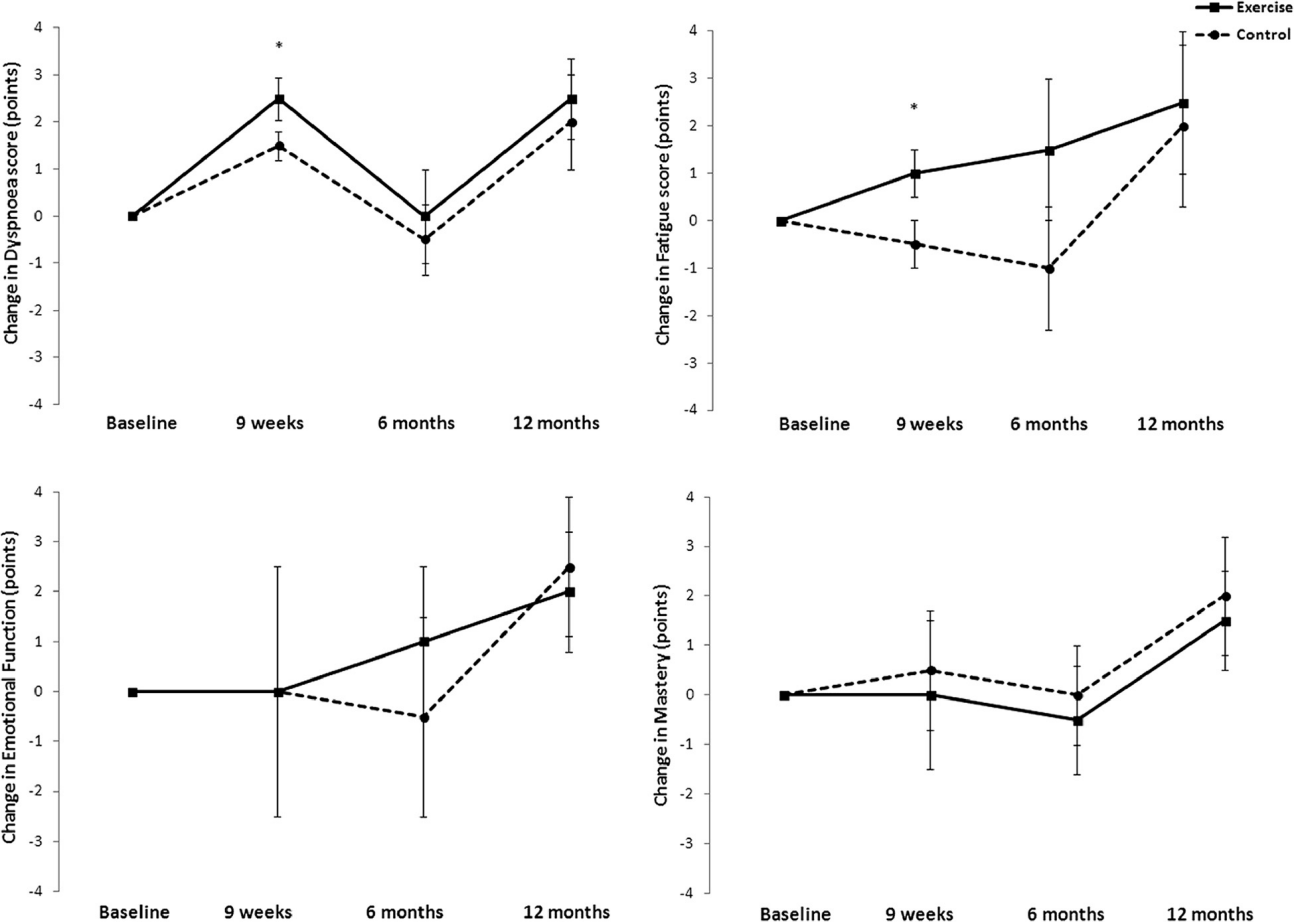
Change in health-related quality of life – Chronic Respiratory Disease Questionnaire domains, Data are mean (95% CI), *p < 0.05, exercise vs control group.

The effect of exercise training on maximal exercise capacity is shown in Figure 2. There was a significant interaction between group and time for ISWD (p = 0.005). The ISWD improved in the exercise group following training, with a mean difference (95% CI) of 62 m (24 to 101 m) compared to the control group. However, this improvement was not sustained at either six or 12 months. For the CRDQ, the exercise group demonstrated a significant reduction in dyspnoea (p = 0.009) and fatigue (p =0.01) compared to the control group immediately following training (Figure 3), but these improvements were not sustained when assessed at six and 12 months. There was no significant change in emotional function or mastery.

Of the secondary outcomes, there was a significant improvement in functional exercise tolerance following training, with a mean change (95% CI) in 6MWD of 41 m (19 to 63 m) compared to the control group, but this improvement was not sustained over the longer term. There were no significant differences between groups in any of the LCQ domains or in anxiety or depression immediately following intervention or at follow up (Table 2).

**Table 2:** Effect of exercise training on cough related quality of life and psychological symptoms

	**Baseline**	**9 weeks**	**6 months**	**12 months**	**p value**
LCQ Physical					
Exercise	5.0 (1.0)	5.3 (0.9)	4.7 (1.4)	4.8 (1.7)	
Control	5.0 (1.0)	5.2 (0.8)	4.8 (1.6)	5.5 (1.1)	0.33
LCQ Psychological					
Exercise	4.9 (1.8)	5.5 (1.3)	5.2 (1.7)	5.6 (1.2)	
Control	5.7 (1.3)	5.8 (1.1)	5.3 (1.8)	6.2 (1.0)	0.11
LCQ Social					
Exercise	5.3 (2.7)	4.6 (1.0)	5.5 (2.2)	3.1 (2.5)	0.98
Control	4.8 (1.1)	4.5 (0.9)	5.3 (1.9)	6.2 (1.2)	
LCQ Total					
Exercise	15.2 (3.4)	15.4 (2.1)	15.4 (1.6)	13.5 (3.1)	
Control	15.5 (1.2)	15.5 (1.9)	15.4 (3.6)	17.9 (2.8)	0.82
HADS Anxiety					
Exercise	4.8 (3.4)	4.6 (3.7)	3.7 (2.9)	3.1 (2.5)	
Control	4.4 (3.1)	4.0 (2.7)	3.7 (3.2)	3.4 (3.5)	0.19
HADS Depression					
Exercise	3.1 (2.9)	3.5 (3.4)	2.7 (2.7)	2.7 (2.7)	
Control	3.5 (2.8)	3.2 (2.2)	3.5 (3.3)	2.9 (3.1)	0.63

Data are mean (SD), p value represents difference between groups.

LCQ – Leicester cough questionnaire; HADS – Hospital anxiety and depression scale.

**Figure 4:**
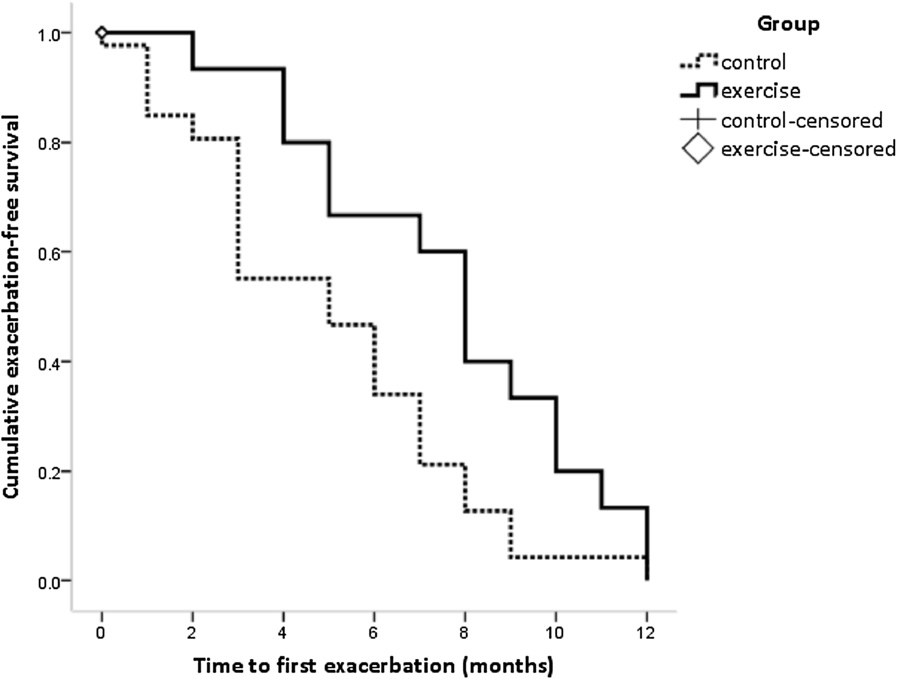
Time to first exacerbation, p = 0.047.

The adherence rate to symptom diary completion was 82% in the exercise group and 77% for the control group. A total of 12 participants in the exercise group and 18 in the control group experienced at least one exacerbation over the 12 months follow up. There was a longer median time to first exacerbation in the exercise group of 8 months (95% CI 7 to 9 months), compared to control group of 6 months (5 to 7 months), with log rank of 0.49 (95% CI 0.01 to 0.97), p = 0.047 (Figure 4). Exercise training reduced the number of exacerbations over 12 months (Table 3), with a trend towards fewer exacerbations requiring antibiotics (p = 0.061). There was no significant difference between groups in the number of days or exacerbation days requiring antibiotics for each exacerbation. The relative risk of exacerbation was 0.69 (95% CI 0.49 to 0.98), indicating there was less risk of exacerbation in the exercise group compared to the control group.

**Table 3:** Number of exacerbations over 12 months (n = 55)

	**Control n = 25**	**Exercise n = 30**	**p value**
Exacerbations	2 (1 – 3)	1 (0 – 2)	0.012
Exacerbations requiring antibiotics	2 (0 – 4)	1 (0 – 2)	0.061
Exacerbation days	10 (2 – 13)	7 (3 – 11)	0.23
Exacerbation days with antibiotics	11 (2 – 15)	7 (2 – 13)	0.36

Data are median (IQR), p value represents difference between groups.

The health utility scores of the AQOL before intervention (baseline) and 12 months following completion of intervention showed a mean (SD) gain in QALYs from 0.74 (0.22) to 0.88 (0.17) in the exercise group and from 0.74 (0.17) to 0.89 (0.11) in the control group. While there was a significant gain in QALYs over 12 months within both groups (both p < 0.001), the mean difference of 0.004 (95% CI −0.08 to 0.09) between groups was not significant.

## Discussion

To date, this is the only trial of exercise training and regular review of ACT in non-CF bronchiectasis which incorporated 12 months follow up. We have demonstrated short term gains in exercise capacity as well as improvement in symptoms of dyspnoea and fatigue, but these benefits were not sustained beyond program completion. With exercise training, the time to first exacerbation was longer and the number of exacerbations over a 12 month period was reduced. There was no benefit in cough related QOL, change in levels of anxiety or depression or greater improvement in QALYs.

This is the first study to show a reduction in the number of acute exacerbations over the 12 months following exercise training and a longer time to first exacerbation. While the reduced exacerbation frequency in the exercise training group should be interpreted with caution, due to the proportion of participants who were unable to complete 12 months follow up, these findings are encouraging and are similar to case series reports [39]. The mechanism by which exercise training reduced exacerbation frequency is unclear. It could be argued that the review of ACT as necessary in the intervention group may account for the difference, but the familiarity of all participants with ACT prior to study enrolment, the lack of change in ACT regimen for the intervention group and the absence of reinforcement over the follow up suggests this is unlikely. Improved immune function has been demonstrated with moderate intensity exercise in healthy elderly individuals [40]; a similar mechanism may be present in this study. In CF, the increase in expiratory flow and promotion of annular airflow during a single session of exercise has been associated with improved mucociliary clearance, although the long term effects are unknown [41]. We did not collect information related to bacterial colonisation, or other markers in inflammation in this study. Given the significant influence of distal airway colonisation upon exacerbation severity and decline in lung function [7,42,43], further examination of the physiological effects of exercise training is needed.

The mean improvement of 64 m in the ISWD and 41 m in the 6WMD is consistent with retrospective findings in this patient population [15,16] as well as those from a comprehensive pulmonary rehabilitation program complemented by ACT [18]. Although the magnitude of increase in ISWD was less in comparison to pulmonary rehabilitation with inspiratory muscle training [17], it suggests that exercise capacity improvement in bronchiectasis can be achieved using training principles similar to those applied in COPD [14,26]. While the level of functional impairment prior to intervention was mild compared to some studies [16,18], the MMRC grades and baseline walking tests were equivalent to other reports of patients included in pulmonary rehabilitation programs [17]. With this magnitude of improvement in patients with mild disease severity and functional limitation, it may be that those with more severe disease and impairment could achieve even greater benefit from this intervention, but this requires further investigation. Our results suggest that the benefits appear to be independent of a comprehensive education program, which has been part of previous work [15,17,18]. While the extent of improvement in 6MWD is similar to the described minimal important difference in COPD [44], this has not yet been defined for bronchiectasis. Therefore the clinical significance of the improvements in exercise capacity requires confirmation.

While exercise training was not associated with a greater improvement in QALYs, in contrast to previous reports in COPD [19], the study was not powered for this outcome and the follow up time was short. Health utility is only one dimension of the cost effectiveness and cost utility of this treatment approach. Further exploration of the economic benefit of this intervention, including measures of cost per QALY [19,35] in bronchiectasis is warranted.

The short-term duration of benefit following exercise training is a common finding in chronic respiratory conditions [14]. Despite monthly telephone follow up and instructions to complete an exercise diary beyond the intervention period, the adherence rate was poor and the absence of a structured exercise program may have impeded the ability to maintain initial improvements. While these results contrast with a retrospective report of pulmonary rehabilitation, which demonstrated that improvement was maintained at 12 months, these effects may have been overestimated, with a significant attrition rate (>50%) in this previous study [15]. The functional decline over time reflects a similar pattern to that observed in COPD and ILD [14,28], despite reports of a slower rate of decline in lung function in bronchiectasis compared to these conditions [45-48]. We found that the 6MWD at 12 months was worse than baseline, suggesting that despite the mild disease evident in this sample, exercise capacity has the potential to decline further over time. Reports in COPD have demonstrated that immediately following pulmonary rehabilitation, an increase in physical activity is evident, but the magnitude is small, with suggestions that a longer duration of treatment is necessary to encourage behavioural change [49-51]. Our results suggest that sustained gains in symptoms and functional capacity may require longer interventions or maintenance programs for people with bronchiectasis.

This is the first prospective study to isolate improvement in dypsnoea and fatigue in people with non-CF bronchiectasis, both key symptoms which contribute to HRQOL [2,3]. The lack of change in psychological symptoms may be due to the few participants with clinically significant anxiety or depression at baseline. Similarly, the well preserved emotional function and mastery at study commencement in both groups, a contrast to COPD [14], may account for the lack of effect on these measures following exercise training. Finally, all participants had long-standing bronchiectasis and the possibility of a greater tolerance of cough-related symptoms, as reflected by the high baseline scores for the LCQ. These clinical factors combined with similar initial instruction in ACT and the consistent regimen during the study in both groups may account for the negligible effect of exercise training on cough-related QOL.

In conclusion, this study demonstrates that a supervised exercise program of eight weeks is associated with short-term improvement in exercise capacity, dyspnoea and fatigue. Exercise training is associated with a reduction in the frequency of acute exacerbations of bronchiectasis over 12 months, however it appears to have minimal effect on cough related QOL. The significance of these findings for long-term prognosis requires further exploration.

## Abbreviations

ACT: Airway clearance techniques; AQOL: Assessment of quality of life; CF: Cystic fibrosis; COPD: Chronic obstructive pulmonary disease; CRDQ: Chronic respiratory disease questionnaire; FEV1: Forced expiratory volume in one second; FVC: Forced vital capacity; HADS: Hospital anxiety and depression scale; HRQOL: Health-related quality of life; HRCT: High resolution computed tomography; ILD: Interstitial lung disease; IQR: Interquartile range; ISWD: Incremental shuttle walk distance; ISWT: Incremental shuttle walk test; LCQ: Leicester cough questionnaire; LOCF: Last observation carried forward; MMRC: Modified Medical Research Council; QOL: Quality of life; SD: Standard deviation; VAS: Visual analogue scale; 6MWD: 6-minute walk distance; 6MWT: 6-minute walk test.

## Competing interests

The authors declare that they have no competing interests.

## Authors’ contributions

Conception and design: AL, CH, NC, SJ, CM, LR, PT, RS, AH. Acquisition of data: AL, CH, NC, SJ, AB. Analysis and interpretation: AL, CH, NC, SJ, CM, AH. Drafting the article for important intellectual content: AL, CH, NC, SJ, CM, AB, LR, PT, RS, AH. All authors contributed to the intellectual content of the manuscript and were consulted for final approval of the submitted version.
